# Trabeculectomy and EX-PRESS Implantation in Open-Angle Glaucoma: An Updated Meta-Analysis of Randomized Controlled Trials

**DOI:** 10.1155/2019/2071506

**Published:** 2019-09-24

**Authors:** Yi Sun, Bowen Zhang, Rouxi Zhou, Tao Wang, Juan Deng

**Affiliations:** ^1^Department of Ophthalmology, Third Affiliated Hospital of Sun Yat-sen University, Guangzhou 510630, China; ^2^Surgical Department, The First Affiliated Hospital of Guangzhou Medical University, Guangzhou 510120, China; ^3^State Key Laboratory of Ophthalmology, Zhongshan Ophthalmic Center, Sun Yat-sen University, Guangzhou 510623, China

## Abstract

**Purpose:**

Accumulating studies comparing the efficacy and safety of trabeculectomy and EX-PRESS implantation in open-angle glaucoma (OAG) report inconsistent findings. Thus, we conducted the updated meta-analysis to clarify the issue.

**Methods:**

Randomized controlled trials (RCTs) were selected through search of databases PubMed, Web of Science, Embase, and the Cochrane Library from their inception up until November 2018. The pooled mean difference (MD) for intraocular pressure reduction (IOPR) and antiglaucoma medication reduction, odds ratio (OR) for operative success, complication, and postoperative intervention was calculated using the random effects model.

**Results:**

8 RCTs were enrolled, including 223 eyes in the EX-PRESS group and 217 eyes in the trabeculectomy group. EX-PRESS device implantation had a better IOPR% at 12 months postoperatively (MD = 8.9, 95% confidence interval (CI) = 2.5–15.3, *P*=0.006). There was no statistically significant difference in the antiglaucoma medication reduction (MD = 6.01, 95% CI = −4.13–16.15, *P*=0.25) and qualified success (*P* > 0.05). Statistically higher complete success at 1 year postoperatively was found in the EX-PRESS group (OR = 3.26, 95% CI = 1.24–8.55, *P*=0.02). EX-PRESS was associated with a lower frequency of increased IOP (OR = 0.15, 95% CI = 0.03–0.93, *P*=0.04) and hyphema (OR = 0.20, 95% CI = 0.05–0.74, *P*=0.02). Less postoperative intervention was needed in the EX-PRESS group (OR = 0.43, 95% CI = 0.20–0.94, *P*=0.04).

**Conclusion:**

For OAG patients, EX-PRESS implantation provided better efficacy in IOP control and complete success at 1 year postoperatively, with fewer increased IOP and hyphema as well as postoperative interventions. EX-PRESS device and trabeculectomy were similar in the qualified success and antiglaucoma medication reduction.

## 1. Introduction

Glaucoma is the leading cause of irreversible blindness worldwide. It is estimated that 6.7 million people will be affected by open-angle glaucoma (OAG) in 2020 [1]. Trabeculectomy is the most widely utilized approach for OAG [2] with success and well-established complications such as shallow anterior chamber [3, 4]. EX-PRESS miniature glaucoma shunt is a nonvalved stainless steel tube, involved more in simpler and faster surgical procedures than conventional trabeculectomy. Now, EX-PRESS is an alternative filtration operation for OAG and gaining popularity.

Many studies were conducted to assess the safety and efficacy of trabeculectomy versus EX-PRESS implantation in management of OAG [5–18], showing controversial conclusions. Two meta-analyses of 4 randomized controlled trials (RCTs) were performed in 2014, and the results showed that both methods provided similar intraocular pressure (IOP) control, but the complete operative success was favorable to Ex-PRESS [19, 20]. However, the conclusion has not been consistently supported by another 6 RCTs published thereafter [6, 9, 10, 13–15]. Some studies confirmed the finding, while others showed similar performance. The results might be different due to the addition of another 6 new studies. Therefore, we performed an updated meta-analysis to further evaluate the efficacy and safety of trabeculectomy vs EX-PRESS device implantation in OAG patients.

## 2. Materials and Methods

The study was performed in accordance with the Preferred Reporting Items for Systematic Reviews and Meta-Analyses (PRISMA) statement ([Supplementary-material supplementary-material-1]).

### 2.1. Search Strategy

The study was registered in PROSPERO database with an ID of CRD42019120540. Databases of PubMed, Web of Science, Embase, and the Cochrane Library were searched from their inception up until November 2018. Details of the search strategies are described in the search strategy file. EndNote software was used to exclude the duplications. Titles and abstracts were screened to subtract obviously irrelevant studies. Full texts were retrieved and appraised for eligibility. Manual search was performed by checking the reference lists of all acquired studies and reviewing articles to identify studies not found by the electronic searches. No language restriction was applied.

### 2.2. Inclusion and Exclusion Criteria

Articles were considered qualified if they fulfilled the following inclusion criteria: (1) participants: OAG patients who could not be controlled with the maximum antiglaucoma medicine; (2) intervention: trabeculectomy versus EX-PRESS implantation; (3) outcomes: at least one of the outcomes of interest discussed below was involved; (4) follow-up time: at least 6 months postoperatively; and (5) publication type: RCT. RCTs without exact raw data available for extraction were excluded. The most recent study was included for successive publications on the same group of patients, but data that were not obtainable from the latest publication were gained from the previously available reports.

### 2.3. Outcome Measurements

For efficacy, the primary outcome was the percentage of intraocular pressure reduction (IOPR%) and antiglaucoma medication reduction. The mean value and standard deviation (SD) of the IOPR% were used directly if they were reported by authors. Otherwise, IOPR and SD_IOPR_ were calculated according to the formulations: IOPR = IOP_baseline_ − IOP_time-point_, SD_IOPR_ = (SD_baseline_^2^ + SD_time point_^2^ − SD_baseline_ × SD_time point_)^1/2^; then the IOPR% and the SD of the IOPR% (SD_IOPR%_) were estimated by IOPR% = IOPR/IOP_baseline_, SD_IOPR%_ = SD_IOPR_/IOP_baseline_ [21]. Calculation of the percentage of antiglaucoma medication reduction was similar to IOPR%. The second outcome was the proportion of patients with complete success and qualified success. Complete success was defined as target endpoint IOP without antiglaucoma medication, while qualified success was defined as target endpoint IOP with or without antiglaucoma medication. The tertiary outcome was the proportion of patients needing postoperative intervention, such as bleb revision. For safety, the outcome was at least one complication, for example, shallow or flat anterior chamber, hyphema, hypotony, and choroidal effusion.

### 2.4. Data Extraction

Data were extracted by two independent reviewers. Discrepancies between the investigators were resolved by discussion to reach a consensus. The discrepancies will be arbitrated by the third reviewer, if necessary. Information collected from each study included the first author's last name, year of publication, location and follow-up time, sample size, antimetabolites, and diagnoses.

### 2.5. Risk of Bias Assessment

Two reviewers separately assessed the risk of bias in each study using the methods described in the Cochrane Handbook for Systematic Reviews of Interventions 5.3. The authors reviewed the studies and allocated a score of “high,” “low,” or “unclear” to the following items: (1) selection bias (sufficient generation of the randomization sequence and allocation concealment?); (2) performance and detection bias (blinding of personnel, participants, and outcome assessors?); (3) attrition bias (incomplete outcome data and how to deal with this?); (4) reporting bias (evidence of reporting outcome selectively?); and (5) other sources of bias (any other potential threats to validity?). Any contradiction was discussed until a consensus was achieved. The advice from the third reviewer will be sought if necessary.

### 2.6. Statistical Analysis

IOP reduction and antiglaucoma medication reduction were regarded as continuous variables with the mean difference (MD) measured. Complication, postoperative success, and intervention were handled as dichotomous variables measured as the odds ratio (OR). All outcomes were reported with a 95% confidence interval (CI). Data were pooled using a random effects model to achieve more conservative estimates [22]. Statistical heterogeneity among the studies was evaluated by calculating a Cochran's Q statistic and an *I*^2^ statistic. *P* < 0.1 in Cochran's Q statistic or an *I*^2^ value greater than 50% exhibits significant heterogeneity [23]. A subgroup analysis stratified by study characteristics was conducted to explore the source of heterogeneity. The analysis was performed using Review Manager 5.3 (the Cochrane Collaboration, Copenhagen, Denmark). A *P* value of less than 0.05 was considered statistically significant.

### 2.7. Sensitivity Analysis and Publication Bias

Subgroup analyses in terms of IOPR, success rate, and complication at different time points were performed. In addition, sensitivity analyses were undergone by omitting one study in each turn to investigate the influence of a single study on the overall pooled estimate. We visually examined asymmetry in funnel plots in order to detect publication biases. Besides, Begg's and Egger's measures should also be calculated.

## 3. Results

### 3.1. Literature Search

Literature search and selection process are summarized in [Supplementary-material supplementary-material-1]. A total of 135 articles were initially enrolled. After removing duplications, the abstracts of the remaining studies were inspected, and 49 articles with possibly relevant trials were further identified in full texts. 8 articles were from three cohorts, respectively [5, 7, 9, 12, 13, 15–17], so the most recent three articles were chosen. Thus, 8 RCTs were eligible after a full-text screening [6, 8–14] and were finally included in the meta-analysis. Kappa statistic between the investigations was calculated based on the formula of the Cochrane Handbook for Systematic Reviews of Interventions, and the value was 0.667.

### 3.2. Characteristics and Quality Assessment of the Included Studies

Characteristics of the included studies are summarized in Table 1. 440 eyes were enrolled: 217 in the trabeculectomy group and 223 in the EX-PRESS group. Quality assessment was conducted on the basis of the Cochrane Handbook for Systematic Reviews of Interventions 5.3. The risks of biases in these studies are demonstrated in [Supplementary-material supplementary-material-1].

### 3.3. Efficacy Analysis

**Figure 1:**
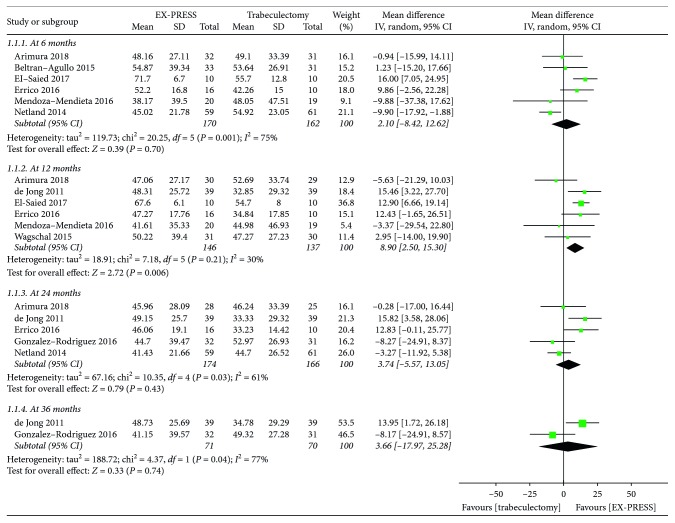
Intraocular pressure reduction (%) from the baseline to time points postoperatively comparing EX-PRESS to trabeculectomy.

**Figure 2:**
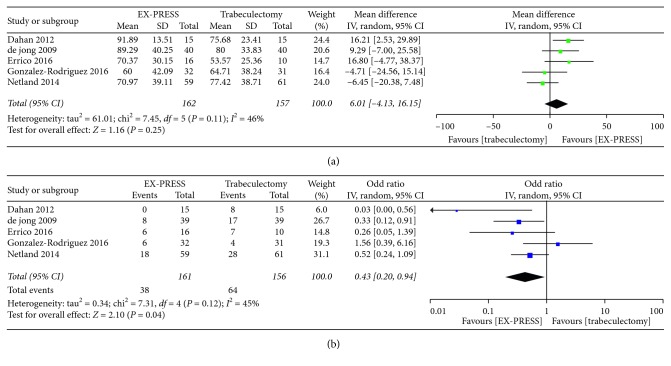
Antiglaucoma medication reduction (%) from the baseline to the endpoint postoperatively (a) and postoperative intervention (b) between EX-PRESS and trabeculectomy.

**Figure 3:**
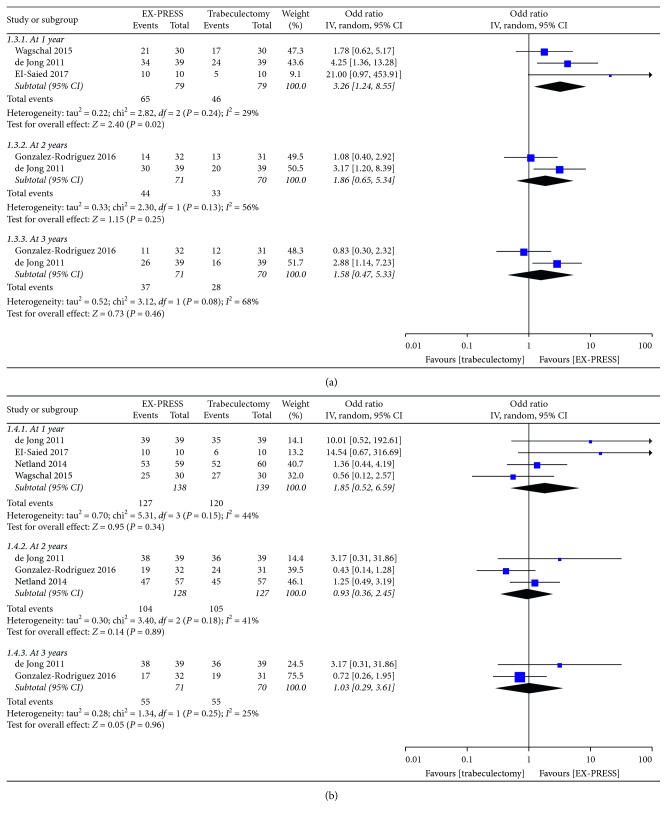
Complete success (a) and qualified success (b) at different time points postoperatively comparing EX-PRESS to trabeculectomy.

The pooled results showed a similar IOPR% postoperatively between the groups except a statistically significant difference favoring EX-PRESS at 12 months (MD = 8.9, 95% CI = 2.5–15.3, *P*=0.006; *P*_heterogeneity_ = 0.21, *I*^2^ = 30%) (Figure 1). Due to the significant heterogeneity among studies on IOPR%, subgroup analysis according to follow-up duration, sample size, publication year, and mitomycin-C concentration was conducted (Table 2). The results showed that follow-up duration and mitomycin-C concentration were the main source of heterogeneity. There was no statistically significant difference between EX-PRESS and trabeculectomy in the percentage of antiglaucoma medication reduction (MD = 6.01, 95% CI = −4.13–16.15, *P*=0.25; *P*_heterogeneity_ = 0.11, *I*^2^ = 46%) (Figure 2(a)). The complete success at 1 year postoperatively was in favor of the EX-PRESS group (OR = 3.26, 95% CI = 1.24–8.55, *P*=0.02; *P*_heterogeneity_ = 0.24, *I*^2^ = 29%) (Figure 3(a)). But both surgical procedures were similar in the qualified success at 1, 2, and 3 years postoperatively (Figure 3(b)).

### 3.4. Safety Analysis

**Figure 4:**
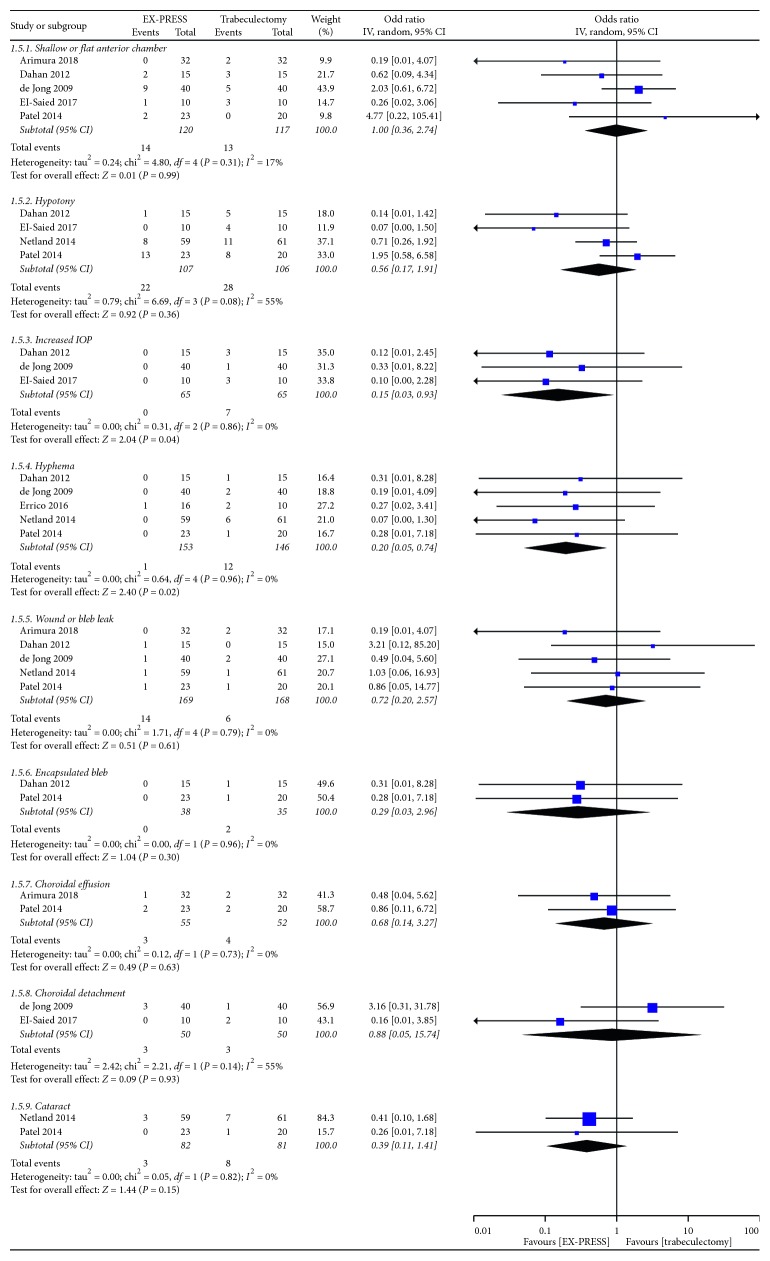
Postoperative complications between EX-PRESS and trabeculectomy.

Postoperative complications between EX-PRESS and trabeculectomy are shown in Figure 4. Shallow or flat anterior chamber, hypotony, hyphema, and bleb leak were the most commonly reported complications. EX-PRESS was associated with a statistically significant lower frequency of increased IOP (OR = 0.15, 95% CI = 0.03–0.93, *P*=0.04; *P*_heterogeneity_ = 0.86, *I*^2^ = 0%) and hyphema (OR = 0.20, 95% CI = 0.05–0.74, *P*=0.02; *P*_heterogeneity_ = 0.96, *I*^2^ = 0%). Less pooled postoperative interventions were required in the EX-PRESS group (OR = 0.43, 95% CI = 0.20–0.94, *P*=0.04; *P*_heterogeneity_ = 0.12, *I*^2^ = 45%) (Figure 2(b)).

### 3.5. Sensitivity Analysis and Publication Bias

To check the sensitivity in each outcome analysis, single study was excluded in turn to evaluate the effect of individual study on the pooled results. Sensitivity analysis was undergone for the outcomes available in more than 3 articles. The percentage of IOP reduction and complete success at 12 months postoperatively, antiglaucoma medication reduction, complications of increased IOP and hyphema, and postoperative additional intervention were altered (data not listed). Publication biases were not checked due to the limited study (<10) involved for each outcome.

## 4. Discussion

Glaucoma is characterized by the progressive defect of the visual field. The goal of glaucoma treatment is to reduce IOP. Among many treatment options, trabeculectomy has been dominating for many years [24]. Due to the associated complications of trabeculectomy, advances have been made to improve the outcome of operation, including the development of EX-PRESS mini shunt implantation. But the efficacy and safety between the both surgeries were still inconsistent despite the meta-analyses was conducted previously. This meta-analysis, updated with 440 eyes in 8 RCTs, showed that for OAG patients, EX-PRESS implantation was better than trabeculectomy in short-term IOP control and complete success, with fewer postoperative complications of increased IOP and hyphema.

The earlier meta-analyses demonstrated that EX-PRESS and trabeculectomy provided similar efficacy in IOP-lowering [19, 20]. But only 4 studies were involved in both meta-analyses, which might be difficult to draw an accurate conclusion. By contrast, raw data on IOPR from more articles could be extracted in our present study, bringing forth relatively convincing results. The pooled analysis revealed that a statistically significant IOPR difference favorable to EX-PRESS existed at 12 months postoperatively, which indicated that the maximum IOP-lowing range of the EX-PRESS filtration device might take place during the early postoperative period. It should be mentioned that pooled analysis of IOPR at 1 year postoperatively by Netland was not conducted due to the unavailable exact IOP data, although with 2-year follow-up [8].

The complete success rate in the EX-PRESS group was higher than that in the trabeculectomy, but the success rate did not differ significantly when antiglaucoma medication was used. One of the possible reasons might be the inconsistent definition of complete success and qualified success in the enrolled publications. In most studies [7, 9, 11], complete success was defined as IOP 5∼18 mmHg and 20% reduction from the baseline without antiglaucoma medications, while qualified success was the same criteria mentioned above with or without antiglaucoma medications. The difference among other definitions mainly lies in the IOP values irrespective of medication/surgery interventions added or not [8, 10, 14]. Moreover, it was judged that data on success rate by Errico et al. [6], Dahan et al. [11], and Mendoza–Mendieta et al. [14] were incorrect or unextracted, so the above studies were not included for analysis. But the hazard ratio 0.27 of complete success and 0.21 of qualified success reported by Dahan et al. was favorable to EX-PRESS [11].

Although providing satisfactory long-term reduced IOP, the trabeculectomy was associated with a high rate of complication. In the current study, increased IOP and hyphema in EX-PRESS were less frequent than in the trabeculectomy group, which showed the safety of EX-PRESS and was possibly associated with fewer procedures needed in EX-PRESS implantation. The finding was somewhat different from the previous meta-analysis. The relatively more complications after trabeculectomy would accordingly need more additional interventions, such as, bleb needling.

Heterogeneity was found in the current study, especially in the Figure 1. The heterogeneity may be caused by clinical and methodological diversity. The subgroup analysis on IOPR% indicated that the heterogeneity between studies mainly stemmed from follow-up duration and mitomycin-C concentration. The source of considerable heterogeneity in complete success among studies was not explored because the accurate complete success data could be extracted from only 3 studies. In addition to the design lacking high-level quality and ununified definition of success rate, other factors such as surgeons' experience, which was difficult to be assessed, may also partially account for the heterogeneity. Moreover, most participants in the enrolled studies included many OAG types instead of the simple POAG. This could be another reason of heterogeneity, and we also tried to verify it. But the exploration was in vain because it was impossible to separate the data completely.

The present study had several potential limitations. First, we can not completely exclude publication biases, which may account for the results of the meta-analysis. Only the studies by Netland et al. [8], Gonzalez–Rodriguez et al. [9], and Dahan et al. [11] were registered with the NCT number, and the study of Arimura et al. [13]was registered with the Japanese equivalent. Those studies, while begun as randomized trials, may have represented those having a “positive result,” and thus were submitted for publication, while other similar studies or “negative” studies may not have been submitted or accepted for publication, which may have failed to be included. Second, the heterogeneity may result from different types of OAG, surgeon's experience, varied mitomycin-C concentration, and exposure time, as well as ununified criteria of success rate. For example, not all subjects enrolled in the included RCTs were POAG. Other OAG types like pseudoexfoliation glaucoma, pigmentary glaucoma, and secondary OAG were also involved. Third, some of the included studies were carried out with small sample size, inadequate allocation concealment, or no double blinding. These factors may have a potential influence on the results. Fourth, analysis at certain time points was not performed as only one trial was involved. For example, operate success at 4 and 5 years postoperatively was reported only by de Jong et al. [12] and IOPR at 9 months and 30 months postoperatively was described only by Arimura et al. [17] and Dahan et al. [11], respectively. So, the long-term efficacy with follow-up more than 3 years between both procedures remained indefinite. Fifth, sensitivity analyses of some outcome measures were not stable. Therefore, conclusions drawn from the pooled results should be interpreted with caution.

In spite of various limitations, our study is still clinically useful because it shows in OAG patients, EX-PRESS filtration device results in better IOP control and complete success 1 year postoperatively and fewer increased IOP and hyphema as well as postoperative interventions in comparison with trabeculectomy. EX-PRESS and trabeculectomy are similar in qualified success and antiglaucoma medication reduction. In the future, more large-scale, well-designed RCTs with longer follow-up should be urgently warranted to determine whether EX-PRESS can substitute trabeculectomy. Their cost benefits and the long-term risk of glaucoma shunt extrusion should also be considered.

## Figures and Tables

**Table 1:** Characteristics of the included randomized clinical trials.

First author (year)	Location	No. of eyes (EX/Tr)	Type of glaucoma	Antimetabolity	Follow-up (m)
Errico (2016) [6]	Italy	16/10	Secondary OAG	Mitomycin-C 0.1 mg/mL, 5 minutes	24
Netland (2014) [8]	USA	59/61	POAG, PXFG, PG	Mitomycin-C 0.25 mg/mL, 1-2 minutes	24
Gonzalez–Rodriguez (2016) [9]	Canada	32/31	OAG	Mitomycin-C 0.4 mg/mL, 2 minutes	36
El-Saied (2017) [10]	Egypt	10/10	Secondary OAG	Mitomycin-C 0.4 mg/mL, 2 minutes	12
Dahan (2012) [11]	Israel	15/15	POAG	Mitomycin-C 0.05%, 1 minute	30
de Jong (2011) [12]	France	39/39	POAG, PXFG, PG	Mitomycin-C 0.02%	60
Arimura (2018) [13]	Japan	32/32	POAG, XFG	Mitomycin-C 0.4 mg/mL, 4 minutes	24
Mendoza–Mendieta (2016) [14]	Mexico	20/19	POAG, PXFG, PG, steroid-induced, traumatic	Mitomycin-C	19

EX: EX-PRESS; Tr: trabeculectomy; POAG: primary open-angle glaucoma; PXFG: pseudoexfoliation glaucoma; PG: pigmentary glaucoma; MMC: mitomycin-C; min: minute; m: month.

**Table 2:** Subgroup analysis of IOPR% from the baseline to the endpoint comparing trabeculectomy and EX-PRESS.

Group	No. of studies	MD			Test for heterogeneity			Test for overall effect	
		Estimate	Lower	Up	*χ*2	*I* ^2^ (%)	*P*	*Z*	*P*
All	8	3.60	−3.11	10.31	14.49	52	0.04	1.05	0.29
Follow-up									
≤24 months	5	5.23	−3.50	13.96	10.96	64	0.03	1.17	0.24
>24 months	3	0.23	−9.15	9.60	1.82	0	0.40	0.05	0.96
Sample size (each group)									
≤30	4	11.52	6.14	16.89	2.75	0	0.43	4.20	<0.0001
>30	4	−1.37	−7.46	4.73	2.10	0	0.55	0.44	0.66
Publication year									
Before 2014	3	−0.56	−7.44	6.31	1.43	0	0.49	0.16	0.87
After 2014	5	5.72	−3.00	14.44	7.91	49	0.09	1.29	0.20
Mitomycin-C concentration									
<0.4 mg/ml	3	4.14	−5.51	13.78	4.42	55	0.11	0.84	0.40
≥0.4 mg/ml	4	2.54	−9.38	14.46	7.60	61	0.05	0.42	0.68
Unknown	1	−3.37	−29.54	22.8	—	—	—	0.25	0.80

## Data Availability

The data used to support the findings of this study are available from the corresponding author upon request.

## Supplementary Materials

Supplementary Materials Supplementary Figures: the file mainly describes the flow diagram for the literature search and selection process as well as the risk of bias assessment of the included RCTs. Supplementary Table: PRISMA checklist.
