# Supplementary material for: Trabeculectomy and EX-PRESS Implantation in Open-Angle Glaucoma: An Updated Meta-Analysis of Randomized Controlled Trials
Source: J Ophthalmol. 2019 Sep 24;2019:2071506. doi: 10.1155/2019/2071506 (PMC6778870; doi:10.1155/2019/2071506)
Supplement: Supplementary Materials — Supplementary Figures: the file mainly describes the flow diagram for the literature search and selection process as well as the risk of bias assessment of the included RCTs. Supplementary Table: PRISMA checklist. [file 2071506.f1.zip › 2071506.f1/Supplemental Figures.docx]

**Included**

**Eligibility**

**Screening**

**Identification**

Records identified through database searching (n=135)

Records screened(n=88)

Records excluded after abstract review (n=39)

Full-text articles assessed for eligibility(n=49)

Full-text articles excluded (n=41):

●Non-RCT: n=4

●Meeting abstract, editorial, reply, protocol, meta-analysis or review: n=30

●Exact raw data unavailable: n=2

●Article from the same trials: n=5

Studies included in quantitative synthesis (n=8)

Records after duplicates removed(n=88)

Studies included in quantitative synthesis (meta-analysis) (n=8)

Figure S1. Flow diagram for the literature search and selection process.


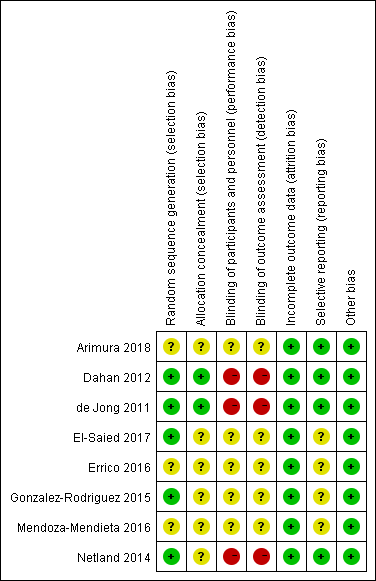


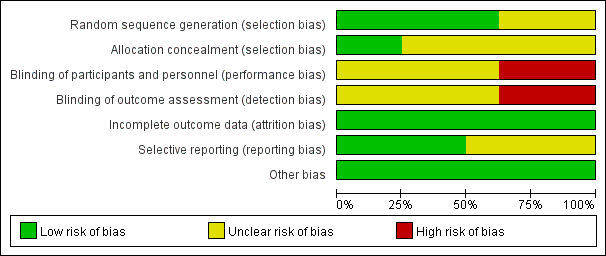


Figure S2. Risk of bias assessment of included RCTs.
