# Supplementary material for: Trabeculectomy and EX-PRESS Implantation in Open-Angle Glaucoma: An Updated Meta-Analysis of Randomized Controlled Trials
Source: J Ophthalmol. 2019 Sep 24;2019:2071506. doi: 10.1155/2019/2071506 (PMC6778870; doi:10.1155/2019/2071506)
Supplement: Supplementary Materials — Supplementary Figures: the file mainly describes the flow diagram for the literature search and selection process as well as the risk of bias assessment of the included RCTs. Supplementary Table: PRISMA checklist. [file 2071506.f1.zip › 2071506.f1/Search Strategy.doc]

**Search strategy for Cochrane Library:**

#1 "random*" or allocation or "random allocation" or placebo or single blind or double blind or "randomized controlled trial*" or RCT or "clinical trial*"

#2 randomized controlled trial:pt or clinical trial:pt

#3 trabeculectomy

#4 EX-PRESS

#5 glaucoma

#6 #1 and #2 and #3 and #4 and #5

**Search strategy for Pubmed:**

#1 "Single-Blind Method"[Mesh] OR "Double-Blind Method"[Mesh] OR "Randomized Controlled Trials as Topic"[Mesh] OR "Randomized Controlled Trial" [Publication Type] OR "Intention to Treat Analysis"[Mesh] OR "Controlled Clinical Trials as Topic"[Mesh] OR "Clinical Trials as Topic"[Mesh] OR "Clinical Trial" [Publication Type] OR randomized controlled trial[Publication Type]

#2 "random*"[Text Word] OR allocation [Text Word] OR "random allocation"[Text Word] OR placebo[Text Word] OR single blind[Text Word] OR double blind[Text Word] OR "randomized controlled trial*"[Text Word] OR RCT[Text Word]

#3 #1 OR #2

#4 trabeculectomy

#5 EX-PRESS

#6 glaucoma

#7 #3 and #4 and #5 and #6

**Search strategy for web of science:**

#1 TS= ("random*" OR allocation OR "random allocation" OR placebo OR single blind OR single blind method OR double blind OR double blind method OR "randomized controlled trial*" OR "randomised controlled trial*" OR "RCT" OR "clinical trial*")

#2 TS= (trabeculectomy)

#3 TS= (EX-PRESS)

#4 TS= (glaucoma)

#5 #1 AND #2 AND #3 AND #4

**Search strategy for EMBASE:**

#1 'randomization'/exp OR 'placebo'/exp OR 'placebo effect'/exp OR 'single blind procedure'/exp OR 'double blind procedure'/exp OR 'randomized controlled trial'/exp OR 'randomized controlled trial (topic)'/exp OR 'controlled clinical trial'/exp OR 'controlled clinical trial (topic)'/exp OR 'clinical trial'/exp OR 'clinical trial (topic)'/exp

#2 random*:ab, ti OR allocation:ab,ti OR "random allocation":ab,ti OR placebo:ab,ti OR single blind:ab,ti OR double blind:ab,ti OR randomised controlled trial*:ab,ti OR randomized controlled trial*:ab,ti OR RCT:ab,ti OR clinical trial*:ab,ti

#3 #1 OR #2

#4 trabeculectomy

#5 EX-PRESS

#6 glaucoma

#7 #3 AND #4 AND #5 AND #6
